# Porous-coated metaphyseal sleeves and MBT implant for severe bone loss in revision total knee arthroplasty: a mean 2.4-year follow-up

**DOI:** 10.1186/s42836-020-00031-x

**Published:** 2020-04-20

**Authors:** Yushun Wu, Eryou Feng, Yiyuan Zhang, Feitai Lin, Liqiong Lin, Zhanglai Li, Lili Xiao

**Affiliations:** 1 Department of Arthrosis Surgery Fuzhou Second Hospital Affiliated to Xiamen University, Fuzhou, China; 2 Fujian University of Traditional Chinese Medicine, Fuzhou, China

**Keywords:** Total knee arthroplasty (TKA), Porous-coated metaphyseal sleeves, Bone loss, Revision

## Abstract

**Background:**

Porous-coated metaphyseal sleeves are designed to fill bone loss and facilitate osseo-integration when bone loss occurs during revision total knee arthroplasty (TKA). The purpose of this paper was to evaluate the clinical and radiographic outcomes of porous-coated metaphyseal sleeves for severe bone loss in revision TKA.

**Methods:**

Form December 2014 to March 2018, we retrospectively analyzed 36 patients receiving revision TKAs. They had Anderson Orthopaedic Research Institute (AORI) Type II and III tibial bone loss and were treated with metaphyseal sleeve. The patients were followed up for a mean time of 28.5 months. The Knee Society Score (KSS), the Hospital for Special Surgery (HSS) Knee Score, Visual Analog Scale (VAS) score and the range of motion (ROM), radiographic findings of sleeve osteo-integration were also recorded. The paired *t* test was used to compare the KSS, the HSS knee score and VAS score before and after the revision TKAs. A value of *P* < 0.05 was considered statistically significant.

**Results:**

Thirty-six patients had complete clinical and radiographic data. At the final follow-up (mean: 28.5 months), significant improvements in knee range of motion, KSS, HSS score and VAS score were observed postoperatively (*P* < 0.001 for all). No aseptic implant fixation failure occurred. Radiographic reviews at the final follow-up revealed that components were stable without occurrence of component migration or clinically significant osteolysis.

**Conclusions:**

This short-term retrospective study illustrated that porous-coated metaphyseal sleeves were useful in revision TKA, with a low rate of intraoperative complications, excellent osteo-integration and stable fixation.

## Introduction

The clinical efficacy of knee arthroplasty for the treatment of end-stage knee osteoarthritis has been recognized [1]. In many countries, the number of revision surgeries is expected to increase [2, 3]. With virtually all knee revisions, bone loss is one of the problems that need to be addressed intraoperatively, and a firm fixation of the revision implant in damaged bone mass can be challenging [4, 5].

The reconstruction options for bone loss include bone cement filling, screw-reinforced bone cement, metal reinforcement, autologous bone grafting, allogeneic bone grafting, and the porous cones and porous-coated sleeves [4, 6–9]. Each technique has advantages and shortcomings, and the results vary. So far, the best strategy for reconstructing significant metaphyseal bone loss during TKA revisions has not been established.

In recent years, the modular cementless metaphyseal fixation has been successfully introduced into revision TKA [10, 11], and porous-coated sleeves reconstruction has been drawing mounting attention. The concept of the sleeve is based on Wolf’s law (1896), which states that stress is distributed to the metaphysis to stimulate bone growth toward the sleeve. By this fixation close to the joint, the rod achieves alignment and serves as a guide, as well as enhances the osseo-integration of the cuff during the first 3 months. This prosthesis prevents potential complications, such as cement blockage, wedge enhancement and disease, enables transmission of the allografts and acts as a stable scaffold for joint reconstruction [12–14]. However, to the best of our knowledge, only a few studies of sleeve prostheses for patients in western countries have been published, involving a limited number of patients and a relatively short follow-up time. Moreover, studies were scanty concerning use of the porous-coated metaphyseal sleeves and MBT implants in revision TKA in Asians, especially in Chinese. The purpose of this study was, therefore, to evaluate the clinical and radiographic outcomes of porous-coated metaphyseal sleeves used for severe bone loss in Chinese people during revision TKA. Our hypothesis was that metaphyseal sleeves could enhance radiographic signs of bone ingrowth and lower the short-term revision rate.

## Methods

From December 2014 to March 2018, a retrospective study was conducted in 36 consecutive patients (involving 36 knees) with AORI II or AORI III bone loss who underwent a revision TKA using a press-fit tibial and/or femoral porous-coated metaphyseal sleeve. The study was approved by the institutional ethics committee of Fuzhou Second Hospital Affiliated to Xiamen University, and written informed consent was obtained from each subject [15].

By retrospectively reviewing all medical records and operative reports, data on the age, gender, weight, height, body mass index (BMI) were collected (Table 1). The primary diagnosis leading to revision TKA included aseptic loosening, prosthetic joint infection, stiff knee, instability, peri-prosthetic fracture and polyethely wear (Table 2). The most common indications for the index revision included aseptic loosening (16 patients, 44.44%), followed by infection (5 patients, 13.8%) and pain or stiffness (5 patients, 13.8%). In addition, preoperative radiographs and intraoperative findings were assessed to classify tibial and femoral bone loss according to the Anderson Orthopaedic Research Institute (AORI) bone defect classification (Table 3**)**. The Knee Society Score (KSS) [16], the Hospital for Special Surgery (HSS) Knee Score [17], and range of motion (ROM) [18] were also collected.

**Table 1:** Preoperative demographics

Gender distribution	21 females/15 males
Age	66 (56–82)
Body weight	73.8 (63.2 ~ 88.7)
Height	163.2 (153 ~ 181)
BMI	27.71 (17.9 ~ 45)
Knee deformities	26 varus knees/10 valgus knees

**Table 2:** Causes for revision TKA

Loosening	16	44.44%
Infection	5	13.80%
Pain/Stiffness	5	13.80%
Osteolysis	4	11.10%
Instability	3	8.30%
Peri-prosthetic fractures	2	5.60%
Polyethely wear	1	2.70%

**Table 3:** Bone loss against AORI classification

AROI Grade	Tibia	Femur	Both tibia and femur
IIA	6	3	3
IIB	8	2	2
III	5	3	4

*AORI* Anderson Orthopaedic Research Institute

All the revision procedures were performed by the same senior surgeon (Dr Eryou Feng, MD). The primary prostheses were adequately exposed via medial parapatellar approach and were subjected to further exposure whenever necessary. The subjects included three cases of tibial tubercle osteotomy and three cases of quadriceps snip. All primary prostheses were removed with special instruments. And then the bone defect on tibial and femoral sides was evaluated against the AORI classification system.

The tibia bone resection was routinely performed and afterward the bone was prepared with hand reaming and broaching. Then, the right sleeve and stem size was assessed with corresponding instruments and trials. If the contacting area between sleeve surface and the metaphyseal host bone was more than the two thirds of the circumference of the porous-coated sleeve, tibia stem was not used.

After the tibia trials, including tibia tray, sleeve and stem were assembled and implanted, and the femoral side was prepared with the similar technique. The distal femur valgus angle was generally 5 degrees. The femoral prosthetic rotation was checked by using the gap balance technique. The joint line was confirmed by the distance between the distal femoral surface and the superior medial femoral condyle. The range of motion of knee joint was confirmed, including full extension and 120° flexion at least. The patellar track was checked and optimized by lateral retinacular release.

All patients received porous-coated metaphyseal sleeves and MBT SIGMA TC3 implants (DePuy Synthes, Raynham, Massachusetts, United States). Nineteen sleeves were used on the tibial side; 8 sleeves on the femoral side; 9 sleeves on both sides. Sleeves with stems were employed in 9 knees.

All the patients received the similar rehabilitating procedure post-operation, including infection prophylaxis, anticoagulant therapy, the extension and flexion motion and the quadriceps strength training. The knee flexion in patients who underwent tibia tubercle osteotomy was limited to less than 60° during the first 6 weeks after operation.

All patients were followed up for 1, 3, 6 month(s) after the operation. Afterwards, they were followed up annually. Knee joint function was assessed in terms of KSS, HSS and VAS scores. Radiographic assessments were performed for each patient at 3, 6 month and then on yearly basis.

The paired *t* test was conducted by using SPSS 22.0 software package (IBM Software Group, Armonk, New York), and *P*-values less than 0.05 were considered statistically significant.

## Results

Of the 36 patients who underwent revision TKA utilizing a porous-coated metaphyseal sleeves and MBT implant. Two patients deceased at time of contact, 1 was lost to follow-up, 2 patients refused to take part in the study. As a result, the final sample size for clinical evaluation consisted of 31 cases. All 31 patients were available for clinical and radiographic review.

The series included 19 women and 12 men, who had a mean body mass index of 27.71 (range 17.9–45), and a mean age of 66 (range 56–82). Twenty-three knees had varus and 8 had valgus deformity. All patients were followed up for an average of 28.5 (12 to 42) months.

During the follow-up, prosthetic joint infection recurred in 2 patients, who required irrigation and debridement, polyethely exchange and components retention and antibiotic treatment. There was no infection recurrence during subsequent follow-up.

Five cases needed evacuation of hematoma identified by ultrasound after continuous wound drainage. Infection was ruled out on the basis of polymorphonuclear percentage and serum inflammtary index.

Eight patients complained of patellar crepitus but they chose not to receive further intervention.

No other complications were noted, such as insability, stiff knee and flexion contracture.

**Fig. 1:**
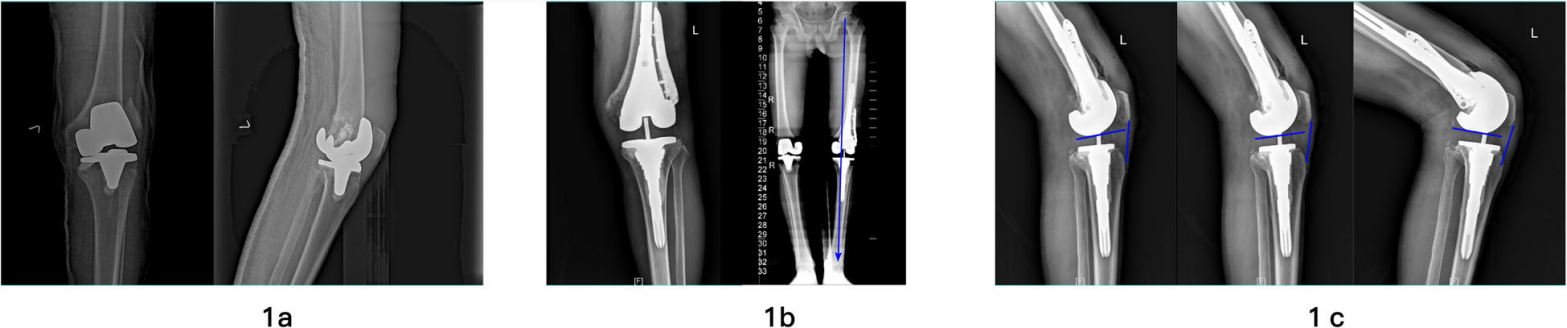
**a** Radiographs of a 68-year-old male who underwent revision TKA for aseptic femoral loosening and distal femoral fractures associated with an AORI type 3 femoral defect. **b** Postoperative full-length standing anteroposterior radiographs demonstrated the the knee was well-aligned and prosthesis was well-fixed. **c** Lateral views of the knee revealed no patella baja

Compared with the preoperative findings, a latest follow-up showed that the mean KSS improved from 33.5 points (range 12–79) to 86.2 points (range 45–99) (*P* < 0.001); the mean HSS score rose from 46.32 points (range 16–82) to 77.6 points (range 32–98) (*P* < 0.001); mean postoperative flexion increased from 59.9° (range 43°-115°) to a mean of 105.1°(range 55°-130°) (*P* < 0.001); mean postoperative VAS score dropped from 6.82 (range 3–10) to mean 3.09 (range 0–8) (*P* < 0.001) (Table 4). Moreover, the follow-up exhibited no radiographic evidence of loosening or progressive radiolucent lines (Fig. 1).

**Table 4:** Clinical outcomes

Time	KSS Score	HSS Score	ROM	VAS Score
Before operation	34.51	46.68	61.36	6.82
After operation	69.66	65.25	90.37	5.32
One month	73.41	67.90	98.23	5.19
Three months	74.64	70.67	99.35	3.64
Half a year	80.25	77.10	104.52	3.45
Latest follow-up	83.83	78.68	107.81	3.09
***t***	11.44	−10.65	−14.57	7.38
***P***	<0.001	<0.001	<0.001	<0.001

*P* The *p*-values, preoperative findings *vs*. the results of the latest follow-up, *KSS* Knee society score, *ROM* Range of motion, *VAS* Visual Analogue Score, *HSS* Hospital for special surgery knee score, *ROM* Range of motion

## Discussion

The greatest challenges in total knee revision surgery are to achieve a soft tissue balance and to minimize the bone loss to restore the knee joint kinematics [19]. Traditional treatment strategies, relying on cement and metal augmentation, bulk allograft, structural allograft, implant composites and trabecular metal cones or impaction grafting, have been successful, to varying degrees, but there are significant concerns regarding graft resorption, vulnerability to fracture, nonunion, collapse, and graft resorption and the potential risk of disease transmission [15, 20–23]. The metaphyseal implants have been a promising alternative to address bone loss in revision TKA [10, 24].

The stability of the prosthesis during revision surgery is critical. Morgan-Jones *et al. *found that there were three anatomical regions in the femur and tibia that are conductive to prosthesis fixation during revision surgery [25]. Because the zone 1 tends to be destroyed in most revision knees by osteolysis, infection, fracture and severe osteoporosis, and could not be used for initial solid fixation of prosthesis.

The porous-coated metaphyseal titannum sleeve has some obvious advantages.

First, sleeve and stem were used to attain effective fixation in at least zone 2 and 3, so that the prosthesis can obtain good initial stability. Moreover, metal sleeve has a porous coating on the surface, which can achieve good biological fixation after implantation in the metaphyseal [10], thereby potentially improving the long-term survival rate.

Second, the greatest advantage is the proximity of fixture to joint surface, which makes the restoration of the joint line easy. Restoration of the joint line itself is a prerequisite for obtaining good functional recovery and joint stability [26, 27].

Third, with fixation being achieved in region 2, the fixation in area 3 becomes less relevant. As a result, the size of the rod and the proportion of medullary cavity filling can be reduced, which could substantially simplify the surgical procedure and shorten the operation time [28, 29].

In our series of revision cases, because the vast majority of tibial and femoral bone surface was destroyed, so most of the bone defect was in the range from type II to type III according to AORI classification. For the type II defect at tibial or femoral side, at least one of the tibial platform or femoral condyle was intact and the contact area between the titanium sleeve and host bone was more than two thirds of the circumference of metaphyseal. Therefore, the sleeve was used without cement or cementless stem. If either tibial platform or femoral condye was not intact, titanium sleeve plus canal stem should be utilized for the extensive fixation in zone 2 and zone 3.

Although stem fixation has been used for a long time in revision TKA with satisfactory results, some problems remain, such as pain at the tip of the stem and difficulty in positioning the stem itself if the femoral and tibial canal were not straight [21]. In our series, 22 sleeves were used without canal stem. The metaphyseal bone was not intact, but stepped titanium sleeve could achieve sufficient press-fit and the solid fixation in zone 2. Our short-term results showed that no sleeve-loosing-related complications developed. And the ROM, KSS, HSS and VAS scores were significantly improved. These results were consistent with those reported by Giacomo* et al*. [30], who performed 46 revision TKAs with sleeve alone either in femoral or tibial side or both. A 37-month follow-up showed that excellent ROM and good clinical results were accomplished in terms of KSS score and WOMAC scores. Their results were further supported by our study. Moreover, there were no signs of implant loosening.

This study had several limitations. This study was of retrospective nature. It didn’t compare its results with other knee revision methods over the same period. The follow-up period lasted for 28.5 (12 to 42) months on average, and a long-term follow-up studies are warranted before a definitive conclusion can be reached.

## Conclusions

Sleeve and MBT implant reconstruction in total knee arthroplasty can repair AORI type II and type III bone loss, increase knee stability, restore the joint line and the soft tissue balance, and facilitate surgical operation. The success rate of revision surgery is high and the short-term clinical results are satisfactory.

## Data Availability

This study was carried out in the Fuzhou Second Hospital affiliated to Xiamen Universitythe (No. 47, Shangteng Road, Cangshan District, Fuzhou, China). The datasets used and/or analyzed during the current study are available from the corresponding author on reasonable request.

## Abbreviations

TKA: Total knee arthroplasty
AORI: Anderson orthopaedic research institute
KSS: Knee society score
ROM: Range of motion
VAS: Visual Analog Score
HSS: Hospital for special surgery knee score
BMI: Body mass index
